# DESIGN OF THE MYECHILD TRIAL, AN INTERNATIONAL RANDOMISED PHASE III CLINICAL TRIAL IN CHILDREN WITH ACUTE MYELOID LEUKAEMIA INCORPORATING AN EMBEDDED DOSE FINDING STUDY

**DOI:** 10.1186/1745-6215-16-S2-O57

**Published:** 2015-11-16

**Authors:** Aimee Houlton, Brenda Gibson, Keith Wheatley

**Affiliations:** 1 Cancer Research UK Clinical Trials Unit (CRCTU), University of Birmingham, Birmingham, UK; 2 Royal Hospital for Sick Children, Glasgow, UK

## 

Acute Myeloid Leukaemia is a rare disease in children but is a significant cause of childhood cancer mortality. This makes running effective clinical trials in the area very difficult.

This trial will test strategies in both induction and consolidation for their value in improving survival by evaluating treatments in four randomised comparisons. The embedded dose finding study (EDFS) aims to identify the optimum dose of Gemtuzumab ozogamicin that can be safely combined with induction chemotherapy, which will then form part of the induction randomised comparisons. A rolling design is applied to the EDFS to reduce the need to pause recruitment between cohorts. Patient's treatment pathways will be determined by their cytogenetics, molecular characteristics and morphological response.

700 patients will be randomised over 6 years. While randomisations 2 and 4 are based on conventional hypothesis testing, randomisation 1 and 3 will use a probability based approach to assess event free survival and relapse free survival. This involves plotting bayesian posterior probability distributions using non-informative priors and observed data to infer the probability that one treatment is better than the other. 280 events are anticipated in randomisation 1, if the observed hazard ratio was 0.89 or better in favour of a particular treatment we could be > 80% sure that this is the more effective treatment. Each randomisation will be analysed in their own right and where appropriate be stratified by previous randomisations. No interaction between treatments is anticipated.

This design will allow effective evaluation of multiple randomised comparisons in small patient populations.

